# Tilapia Skin Peptides Ameliorate Cyclophosphamide-Induced Anxiety- and Depression-Like Behavior *via* Improving Oxidative Stress, Neuroinflammation, Neuron Apoptosis, and Neurogenesis in Mice

**DOI:** 10.3389/fnut.2022.882175

**Published:** 2022-06-02

**Authors:** Yun-Tao Zhao, Haowen Yin, Chuanyin Hu, Jian Zeng, Shilin Zhang, Shaohong Chen, Wenjing Zheng, Mengjiao Li, Leigang Jin, You Liu, Wenjin Wu, Shucheng Liu

**Affiliations:** ^1^Guangdong Province Engineering Laboratory for Marine Biological Products, Guangdong Provincial Key Laboratory of Aquatic Product Processing and Safety, College of Food Science and Technology, Modern Biochemistry Experimental Center, Guangdong Ocean University, Zhanjiang, China; ^2^Department of Biology, Guangdong Medical University, Zhanjiang, China; ^3^State Key Laboratory of Pharmaceutical Biotechnology, Department of Medicine, The University of Hong Kong, Hong Kong, Hong Kong SAR, China; ^4^Institute of Agricultural Products Processing and Nuclear Agricultural Technology, Hubei Academy of Agricultural Sciences, Wuhan, China

**Keywords:** tilapia skin peptides, cyclophosphamide, anxiety- and depression-like behavior, hippocampus, oxidative stress, neuroinflammation, neurogenesis, neuron apoptosis

## Abstract

Anxiety- and depression-like behavior following chemotherapy treatment occurs in cancer patients with high probability and no specific therapeutics are available for treatment and prevention of this complication. Here, tilapia skin peptides (TSP), a novel enzymatically hydrolyzed bioactive peptide mixture, obtained from tilapia (*Oreochromis mossambicus*) scraps, were studied on cyclophosphamide (CP)-induced anxiety- and depression-like behavior in mice. Mice were received intraperitoneal injection of CP for 2 weeks, while TSP was administered for 4 weeks. After the end of the animal experiment, behavioral, biochemical, and molecular tests were carried out. The mice decreased preference for sugar water, increased immobility time in the forced swimming and tail suspension test, and decreased travel distance in the open field test in the Model group, compared with the Control group. Abnormal changes in behavioral tests were significantly improved after the TSP treatment. Additionally, abnormalities on superoxide dismutase, malondialdehyde, glutathione peroxidase were rescued by administration of 1000 mg/kg/d TSP in mice than that of the Model group. TSP has normalized the expression of Iba-1 and the levels of TNF-α and IL-1β in the hippocampus of mice, which indicated that TSP could observably ameliorate neuroinflammatory response in the hippocampus of mice. TSP ameliorated the apoptosis of hippocampal neurons of CA1 and CA3 regions in the TSP group vs. the Model group. The number of doublecortin positive cells was drastically increased by administering 1000 mg/kg/d TSP in mice vs. the Model group. Furthermore, TSP reversed the Nrf2/HO-1 signaling pathway, BDNF/TrkB/CREB signaling pathway, and reduced the Bcl-2/Bax/caspase-3 apoptosis pathway. In conclusion, TSP could restore CP-induced anxiety- and depression-like behavior *via* improving oxidative stress, neuroinflammation, neuron apoptosis, and neurogenesis in mice hippocampus.

## Introduction

Cancer is one of the three major diseases and has brought patients enormous physical and mental pain globally. The number of cancer patients approximately increased by 19.3 million in 2020, globally (1). Chemotherapy is the best choice for controlling disease progression for most cancer patients, but the many side effects cannot be ignored (2). A significant number of cancer patients have psychological problems (depression, anxiety, cognitive impairment, etc.) following chemotherapy treatment that further reduces the quality of lifestyle (3, 4).

Researchers have begun to focus on strategies for preventing or treating anxiety- and depression-like behavior following chemotherapy. Due to the complexity and heterogeneity in the etiology of anxiety and depression, current clinical treatments remain prolonged onset therapeutic effectiveness, adverse effects, and limited effect on one-third of patients (5). When choosing antidepressants and anxiolytics, cancer patients must consider the effects of the drugs on chemotherapy regimens. Cyclophosphamide (CP) is a broad-spectrum anti-tumor pharmaceutical applied routinely for the case of leukemia and solid tumors (6). Phenobarbital and other barbiturates are used clinically as an anxiolytic drug and could promote the enzymatic hydrolysis of cyclophosphamide (CP) into alkylating agents, thereby resulting in readily acute poisoning when used in combination with CP (7). Tamoxifen is metabolized into 4-hydroxytamoxifen and endoxifen through the cytochrome P450 proteins (CYP) pathway to accomplish the chemotherapeutic effect. Antidepressants such as fluoxetine and paroxetine inhibit the CYPD6 enzyme, thereby affecting the effect of chemotherapy (8, 9). Thus, it is urgent to explore a practical therapeutic approach to remedy anxiety- and depression-like behavior following chemotherapy.

Nutritherapy is one of the vital approaches for preventing and intervening with neurobehavioral changes following chemotherapy (10). It is important to replace or supplement drug therapy daily using bioactive substances (11). Accumulating data proves that oxidative stress, neuroinflammation, neuron apoptosis, decreased neurogenesis, and others play a role in chemical neurotoxicity (12, 13). Patients have a significantly increased risk of structural and functional changes in the hippocampus and prefrontal regions of the brain receiving chemotherapy (10, 14). It has been reported that curcumin administration for 14 days could ameliorate CP-induced neuronal oxidative stress in the hippocampus (15). Nerolidol could reverse oxidative stress and neuroinflammation induced *via* CP in the hippocampus (6). Additionally, ganoderic acid could alleviate 5-fluorouracil-induced mitochondrial impairment and neuronal apoptosis (16). Tilapia (*Oreochromis mossambicus*) skin peptides (TSP) have been reported that it has potent antioxidants, anti-inflammation, anti-apoptotic, anti-osteoporosis, anti-skin photoaging, anti-hypertension, and other biological abilities (17–21). Here, we speculate that TSP may play an ameliorative role in chemotherapy-induced nerve injury.

To date, the impact of tilapia skin peptides (TSP) on anxiety- and depression-like behavior following chemotherapy has not been explored. In this study, we established anxiety- and depression-like mice models by injecting CP and tested the effects of TSP on anxiety- and depression-like behavior in mice. In parallel, mechanisms underlying the impact of TSP on anxiety- and depression-like behavior were investigated.

## Materials and Methods

### Reagents

Tilapia skin was donated sincerely by Guolian (Zhanjiang, China). CP was provided *via* Sigma-Aldrich (St. Louis, MO, United States). Superoxide dismutase (SOD) kit, malondialdehyde (MDA) kit, glutathione peroxidase (GSH-Px) kit, and bicinchoninic acid (BCA) kit were purchased from Jiancheng Institute (Nanjing, China). The tumor necrosis factor-alpha (TNF-α) and interleukin 1 beta (IL-1β) commercially mouse ELISA kit were bought from Invitrogen (Carlsbad, CA, United States). The *in situ* TUNEL kit was provided by Roche Group (Basel, Switzerland). Rabbit antimouse ionized calcium-binding adaptor molecule-1 (Iba-1) primary antibody was purchased from Wako (Tokyo, Japan). Mouse antimouse B-cell lymphoma-2 (Bcl-2), caspase-3, and Bcl-2-Associated X (BAX) primary antibodies were donated *via* Santa Cruz (Dallas, TX, United States). Rabbit antimouse kelch-like ECH-associated protein 1 (Keap1) and nuclear factor E2-related factor (Nrf2) primary antibodies were bought from Boster (Wuhan, China). Rabbit antimouse doublecortin (DCX) primary antibody was obtained from Abcam (Cambridge, United Kingdom). Rabbit antimouse heme oxygenase-1 (HO-1), brain-derived neurotrophic factor (BDNF), cAMP response element-binding protein (CREB), and phosphorylated CREB (p-CREB) primary antibodies were bought from Cell Signaling Technology (Danvers, MA, United States).

### Acquisition of TSP

The preparation of TSP follows our previously published papers, which are briefly described as follows (17): tilapia skins were hydrolyzed by neutrase (Ryon Biological, Shanghai, China) at 50°C for 90 min with 0.3% under a pH 7.0, and were hydrolyzed by alcalase (Ryon Biological, Shanghai, China) at 55°C, pH 9.0 for 90 min with 0.3%. Hereafter, the hydrolysis process was interrupted *via* heating at 90°C for 10 min. After passing through a 10000 Da ultrafiltration membrane, TSP was obtained by freeze-drying. The amino acid composition of TSP is as follows (g per 100 g): glycine (22%), proline (10.10%), arginine (8.23%), alanine (7.86%), glutamic acid (6.83%), lysine (3.45%), leucine (3.10%), serine (3.01%), threonine (2.98%), aspartic acid (2.96%), valine (2.16%), phenylalanine (2.10%), isoleucine (1.06%), tyrosine (0.86%), histidine (0.77%), methionine (0.35%) (17). The content of 180–1000 Da molecular weight of TSP was 71.86%. Among them, 34.05% were 500–1000 Da, 37.78% were 180–500 Da, and 5.75% were below 180 Da (17).

### Animals and Treatments

**FIGURE 1:**
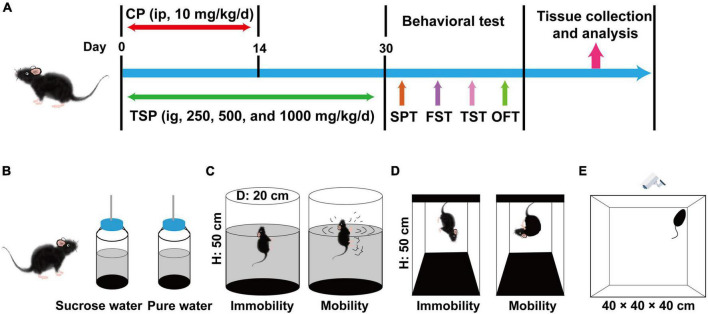
Schematic diagram of the experimental procedures and behavioral experiments. **(A)** Schematic diagram of the experimental procedures. **(B)** Schematic diagram of the SPT experiment. **(C)** Schematic diagram of the FST experiment. **(D)** Schematic diagram of the TST experiment. **(E)** Schematic diagram of the OFT experiment.

Male C57BL/6 mice (aged 8 weeks) were bought from Guangdong Medical Laboratory Animal Center (Guangzhou, China). Five animals were housed per cage in an environment with a temperature of 23 ± 2°C and 40–70% humidity. The mice receive 12 h of light and 12 h of the dark cycle with adequate food and water. Mice were randomly allocated to five groups (*n* = 15 per group): the normal control group (marked as “Control”), CP model group (marked as “Model”), 250 mg/kg/d TSP group (marked as “Model + TSP250”), 500 mg/kg/d TSP group (marked as “Model + TSP500”), and 1000 mg/kg/d TSP group (marked as “Model + TSP1000”). After 1 week of the adaptation period, CP was dissolved in saline solution and administered intraperitoneally (ip) for 2 weeks (10 mg/kg/d) to mice in Model and three doses of TSP groups. The mice in the Control group were received normal saline (1 mL/100 g, ip) for 2 weeks. Three doses of TSP were dissolved in distilled water and treated intragastrically (ig) to mice in three doses of TSP groups for 30 days, respectively. Moreover, the mice in the Control and Model groups were received deionized water (1 mL/100 g, ig) for 30 days. After the behavioral experiments, the hippocampus was collected for testing under pentobarbital sodium anesthesia. Three mice per group were randomly selected and transcardially perfused with PBS and 10% paraformaldehyde during anesthesia. The brain was dehydrated by a sucrose gradient and fixed with the optimal cutting temperature (OCT) compound for analysis. The schematic graph of the experimental process is exhibited in Figure 1A.

### Behavioral Tests

#### Sucrose Preference Test

The schematic diagram of the SPT experiment was shown in Figure 1B. Anhedonia behavior was examined *via* the SPT in accordance with the previous description with favorable modifications (8). Before the experiment, mice were given one bottle of distilled water and one bottle of 1% sucrose water at the same time for 48 h, and the position of the bottles was changed per 12 h. Secondly, mice were isolated and deprived of food and water for 24 h. At the beginning of the test, mice have obtained one bottle of 1% sucrose water and distilled water again, respectively. After drinking for 4 h, the sucrose preference was counted according to the following formula.


$$\text{Sucrose}\;\text{preference}\left(\%\right)=\frac{\text{Sucrose}\;\text{water}\;\text{consumption}}{\text{Total}\;\text{water}\;\text{consumption}}\times 100\%$$

#### Forced Swimming Test

The schematic graph of the FST experiment was exhibited in Figure 1C. The method of FST was appropriately modified based on the previous patterns (22). Prepare several buckets with a diameter of 20 cm and a height of 50 cm, filled with pure water (25 ± 1°C). The mouse was placed in the water for 10 min, and then the mouse was wiped dry to adapt to the environment. The following day, the mice were positioned in the water for 6 min again, and the immobility time (characteristic: float, stop struggling) from the second to the sixth min was recorded by the experimenter.

#### Tail Suspending Test

The schematic diagram of the TST experiment was shown in Figure 1D. The TST experiment was followed by previous research, with appropriate modifications (23). After 30 days of TSP, the mice were suspended 50 cm above the ground using tape placed about 2 cm away from the tip of the tail. The experimenter recorded the immobility time within 5 min when the mouse stopped struggling.

#### Open Field Test

The schematic graph of the OFT experiment was exhibited in Figure 1E. The OFT experiment was carried out according to the published papers (24). Mice were positioned in a white box (40 × 40 × 40 cm) and measured for 5 min, respectively. Before each experiment, 20% alcohol solution was used to remove animal feces, urine, and other odors. The total travel distance was observed using an IR Color CCD camera (Sony, Tokyo, Japan) connected to a computer with SuperMaze Analysis System (XinRuan, Shanghai, China).

### Biochemical Assay

The hippocampal tissue was homogenized in phosphate-buffered saline (PBS, 10% w/v) and centrifuged (10000 g, 12 min) to obtain the supernatant. Additionally, the protein concentration was tested *via* the BCA commercial kit. The resulting supernatant was measured the SOD activity, MDA content, TNF-α level, and IL-1β level using the commercial kit, and the operating procedures followed the instruction manual.

### Immunofluorescence

The immunofluorescence experiment was followed the previous pattern with minor modifications (25). The sections (20 μm thick) were mounted on glass slides and washed for 15 min in PBS containing 0.2% Tween-20 (PBST). The sections were blocked in PBST containing 10% normal horse serum for 1 h and reacted with the primary antibody in blocking buffer overnight. The sections were rewashed for 15 min in PBST to remove unbound antibodies. What is more, the sections were incubated with FITC-conjugated secondary antibodies for 2 h, and the sections were rewashed for 15 min in PBST. The slices were reacted with 4, 6-diamidino-2-phenylindole (DAPI) for 2 min, and the sections were washed for 5 min in PBST. After mounting in glycerol, imaging was performed using a DM2500 Fluorescence Microscope (Leica, Wetzlar, Germany). The mean fluorescence intensity of positive cells was counted *via* the Image-Pro Plus 6.0 software.

### *In situ* TUNEL Analysis

Some DNA structure-specific endonucleases are activated when neurons undergo apoptosis, and the internucleosomal genomic DNA is cut *via* these endonucleases. The exposed 3’-OH of broken DNA can be catalyzed by terminal deoxynucleotidyl transferase (TdT) to bind fluorescein-labeled deoxyuridine triphosphate (dUTP), which an optical microscope can detect. The three brain slides of each sample were randomly selected. The brain slices (20 μm thick) were placed for 20 min at 25°C, fixed in PBS containing 4% paraformaldehyde solution for 25 min. Next, the slices were washed with PBS for 10 min and reacted with proteinase K solution (20 mg/ml) for 15 min, and rinsed with PBS for 10 min. The TdT labeling buffer was dropped to the brain slices, incubated for 60 min at 25°C, and washed with PBS for 30 min. The tissue was reacted with DAPI for 2 min and detected *via* a DM2500 Fluorescence Microscope (Leica, Wetzlar, Germany). All operations were implemented in a dark and wet environment. The number of TUNEL-positive neurons was calculated by the Image-Pro Plus 6.0 software.

### Western Blotting

The Western blotting experiment referred to previous studies and modified it appropriately (26, 27). Trivially, hippocampal tissues were homogenized in 300 μL of radio-immunoprecipitation assay (RIPA) buffer containing phosphatase inhibitor and centrifuged at 10000 g for 12 min. The protein concentration was tested *via* the BCA kit. Proteins were separated by sodium dodecyl sulfate-polyacrylamide gel electrophoresis (SDS-PAGE) and transferred immediately to the polyvinylidene fluoride (PVDF) film, and placed with blocking 5% nonfat dry milk in Tris-buffered saline containing 0.2% Tween-20 (TBST) for 1.5 h. Moreover, The PVDF film was reacted with the primary antibody for 12 h. After TBST washing, the PVDF film was incubated with a horseradish peroxidase-conjugated secondary antibody for 1.5 h. After the rewash, ECL was used for the Western blot experiment. All details were performed at 4°C. The image was processed by the ChemiDoc™ XRS + Imaging System (Bio-Rad, Hercules, CA, United States). Protein bands were normalized relative to the β-actin.

### Statistical Analysis

The results were exhibited using mean ± standard error of measurement (SEM). Experimental data were performed using one-way ANOVA followed by Bonferroni’s *post-hoc* analysis and analyzed *via* the Graphpad Prism 8.0 (GraphPad Software, San Diego, CA, United States). *P*-value < 0.05 was considered statistically significant.

## Results

### Effects of TSP on Cyclophosphamide-Induced Anxiety- and Depression-Like Behavior

**FIGURE 2:**
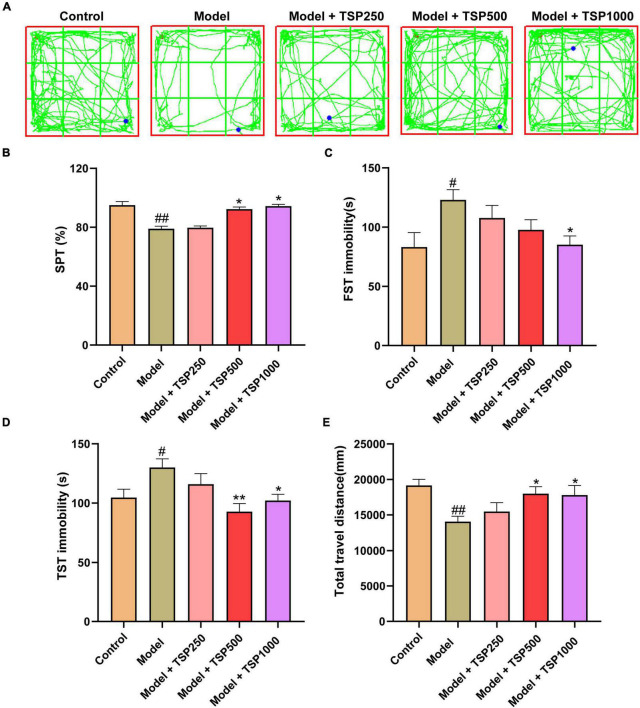
The effects of TSP on anxiety- and depression-like behavior. **(A)** The representative tracks of the OFT experiment. The anxiety- and depression-like behavior was observed *via* SPT **(B)**, FST **(C)**, TST **(D)**, and OFT **(E)** experiments. All data were expressed as mean ± SEM (*n* = 15). ^#^*P* < 0.05, ^##^*P* < 0.01 vs. Control group; *P < 0.05, **P < 0.01 vs. Model group.

In order to research the effect of TSP on CP-induced anxiety- and depression-like behavior in mice, behavioral experiments were investigated. After CP treatment, sugar water preference and total travel distance were markedly reduced, and the immobility time of FST and TST was considerably prolonged (*P* < 0.05, *P* < 0.01, Figure 2) vs. that of the Control groups. These four behavioral conditions were dramatically improved *via* the treatment of different doses of TSP (250, 500, and 1000 mg/kg/d), showing a dose-dependently manner (*P* < 0.05, *P* < 0.01, Figure 2).

### Effects of TSP on Oxidative Stress

**FIGURE 3:**
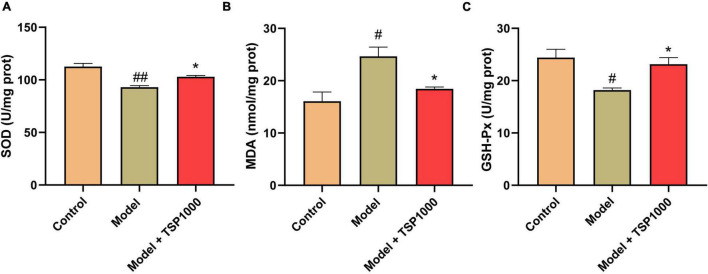
The effects of TSP on oxidative stress. The SOD activity **(A)**, MDA content **(B)**, and GSH-Px **(C)** levels were tested in the hippocampus. All data were expressed as mean ± SEM (*n* = 5). ^#^*P* < 0.05, ^##^*P* < 0.01 vs. Control group; *P < 0.05 vs. Model group.

In order to research the possible mechanism of TSP on anxiety- and depression-like behavior following chemotherapy, mice were injected ip with CP, and a high-dose group was selected for in-depth studies. Compared with that of the Control groups, the SOD activity and GSH-Px level of the hippocampus from the Model group were significantly reduced (*P* < 0.05, *P* < 0.01, Figures 3A,C), and the MDA content was obviously increased (*P* < 0.05, Figure 3B). After treatment with the TSP, SOD activity and GSH-Px level were drastically increased (*P* < 0.05, Figures 3A,C), MDA level was considerably decreased (*P* < 0.05, Figure 3B), indicating that TSP prevents the balance of redox reactions broken by CP in the hippocampus.

### Effect of TSP on Hippocampal Neuroinflammation

**FIGURE 4:**
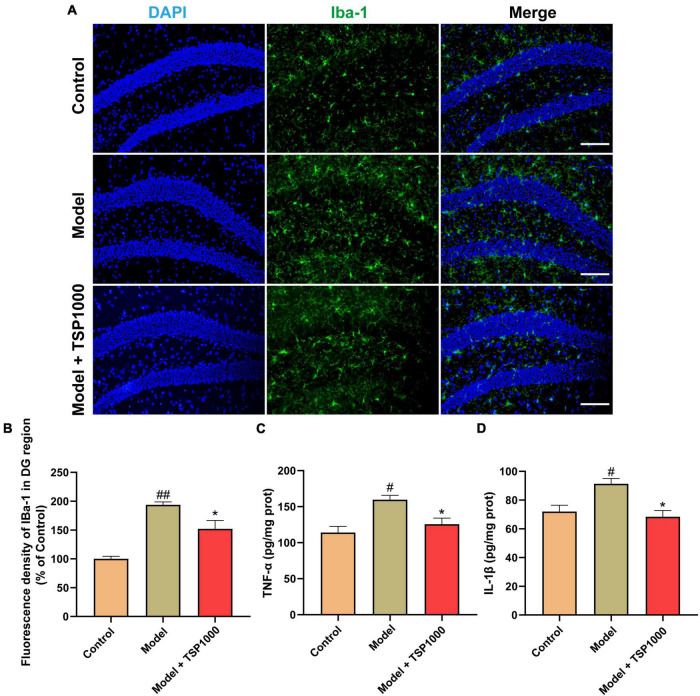
The effects of TSP on hippocampal neuroinflammation. **(A)** The representative staining of activated microglia (Scale bar: 20 μm). **(B)** The fluorescence density of Iba-1 in the DG region was calculated in the hippocampus. The levels of TNF-α **(C)** and IL-1β **(D)** were tested in the hippocampus. All data were expressed as mean ± SEM (*n* = 3). ^#^*P* < 0.05, ^##^*P* < 0.01 vs. Control group; *P < 0.05 vs. Model group.

Hippocampal neuroinflammation is closely related to depression. In order to explore the effects of TSP on hippocampal neuroinflammation, microglia labeled with Iba-1 (Figure 4A) were evaluated *via* immunofluorescence, TNF-α (Figure 4C), and IL-1β (Figure 4C) levels were assessed by ELISA kits in the hippocampus. Compared with that of the Model group, the expression of Iba-1, the levels of TNF-α, and IL-1β in the hippocampus of the mice treated with TSP were markedly reduced (*P* < 0.05, Figure 4). The data showed that TSP could rescue the inflammatory response in the hippocampus.

### Effect of TSP on Hippocampal Neuronal Apoptosis

**FIGURE 5:**
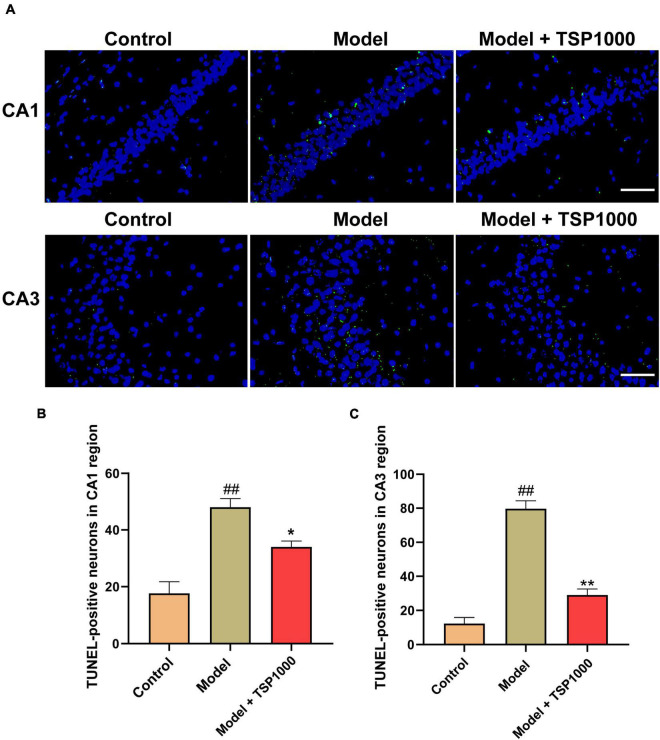
The effects of TSP on neurons apoptosis in the hippocampus. **(A)** Photomicrograph demonstrates *in situ* apoptotic neuron detection by TUNEL stain in the hippocampus of CA1 and CA3 regions (Scale bar: 20 μm). **(B)** The number of TUNEL-positive neurons was quantified. All data were expressed as mean ± SEM (*n* = 3). ^##^*P* < 0.01 vs. Control group; *P < 0.05, **P < 0.01 vs. Model group.

The nuclei of TUNEL-positive neurons (apoptotic neurons) in the hippocampus were stained (Figure 5A). The number of TUNEL-positive neurons in the hippocampus of CA1 and CA3 regions were considerably increased (*P* < 0.01, Figures 5B,C), indicating that CP has a damaging effect on neurons in the hippocampus. TSP-administered mice found that the number of TUNEL-positive cells was markedly reduced vs. that of the Model group (*P* < 0.05, *P* < 0.01, Figures 5B,C).

### Effect of TSP on Hippocampal Neurogenesis

**FIGURE 6:**
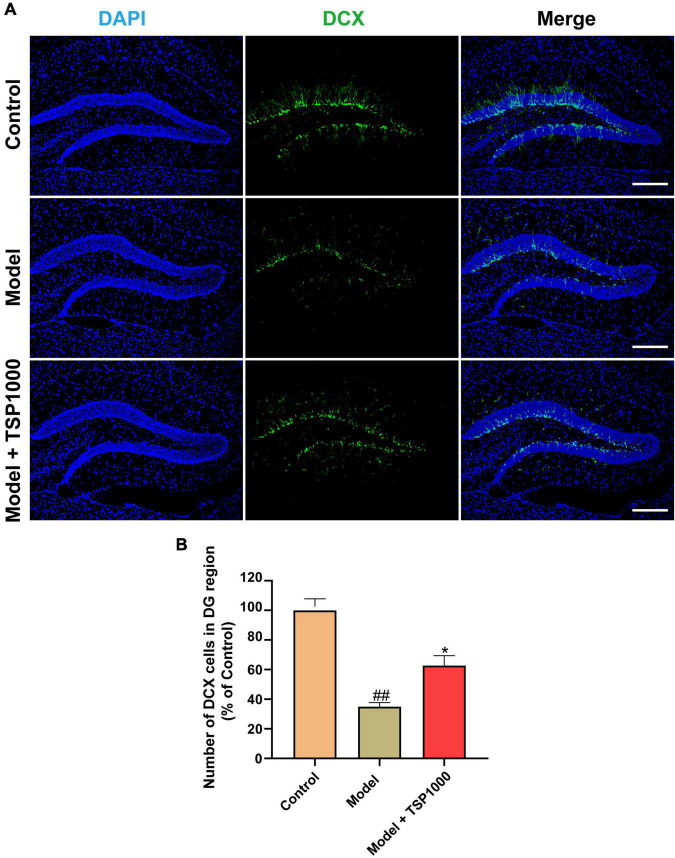
The effects of TSP on neurogenesis in the hippocampus. **(A)** The representative staining of DCX immunofluorescence images in the hippocampus (Scale bar: 100 μm). **(B)** The number of DCX positive cells was calculated. All data were expressed as mean ± SEM (*n* = 3). ^##^*P* < 0.01 vs. Control group; *P < 0.05 vs. Model group.

Accumulated literature has reported that hippocampal neurogenesis exerts an effect on the resistance to depression, so we tested the expression of the neurogenesis marker DCX (Figure 6A). In the Model group, the number of DCX positive cells in the hippocampus of the DG region was considerably lowered vs. that of the Control group (*P* < 0.01, Figure 6B). In contrast, TSP supplements significantly increased the number of DCX positive cells (*P* < 0.05, Figure 6B), compared with that of the Model group.

### Effect of TSP on Nrf2/HO-1 Signaling Pathway

**FIGURE 7:**
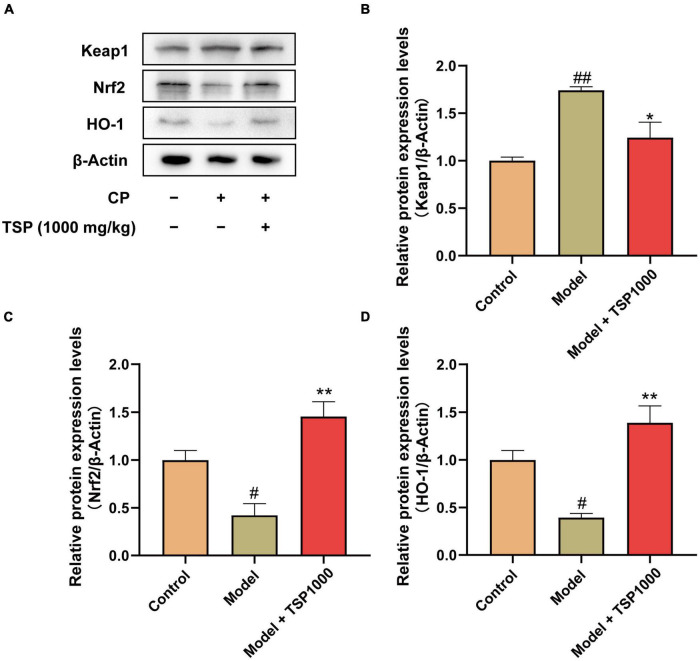
The effects of TSP on the Nrf2/HO-1 signaling pathway. **(A)** The representative images of western blotting results. **(B–D)** Quantification of the protein expression of Keap1, Nrf2, and HO-1. All data were expressed as mean ± SEM (*n* = 3). ^#^*P* < 0.05, ^##^*P* < 0.01 vs. Control group; *P < 0.05, **P < 0.01 vs. Model group.

The Nrf2/HO-1 signaling pathway was investigated as a possible mechanism of oxidative stress (Figure 7). The results exhibited that the protein expression of Keap1 was obviously raised (*P* < 0.01, Figure 7B), and the protein expression of Nrf2 and HO-1 was markedly down-regulated (*P* < 0.05, Figures 7C,D), compared with that of the Control group. TSP-administered mice observed that the Nrf2/HO-1 signaling pathway was obviously activated in the hippocampus (*P* < 0.05, *P* < 0.01, Figures 7B–D).

### Effect of TSP on BDNF/TrkB/CREB Signaling Pathway

**FIGURE 8:**
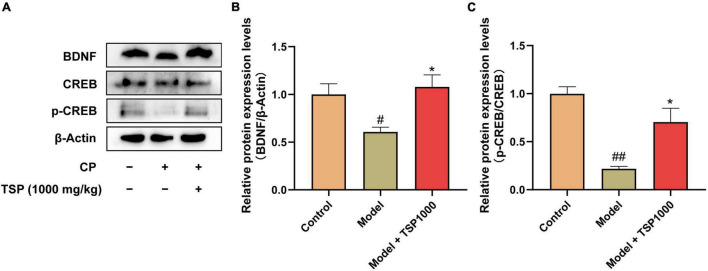
The effects of TSP on the BDNF/TrkB/CREB signaling pathway. **(A)** The representative images of western blotting results. Quantification of the protein expression of BDNF **(B)**, p-CREB/CREB **(C)**. All data were expressed as mean ± SEM (*n* = 3). ^#^*P* < 0.05, ^##^*P* < 0.01 vs. Control group; *P < 0.05 vs. Model group.

The BDNF/TrkB/CREB signaling pathway was evaluated (Figure 8). The results showed that CP significantly suppressed BDNF expression (*P* < 0.05, Figure 8B) and p-CREB expression (*P* < 0.01, Figure 8C) in the hippocampus vs. that of the Control group. 1000 mg/kg-dose TSP administration restored these changes drastically (*P* < 0.05, Figures 8B,C).

### Effect of TSP on Bcl/Bax/caspase-3 Apoptosis Pathway

**FIGURE 9:**
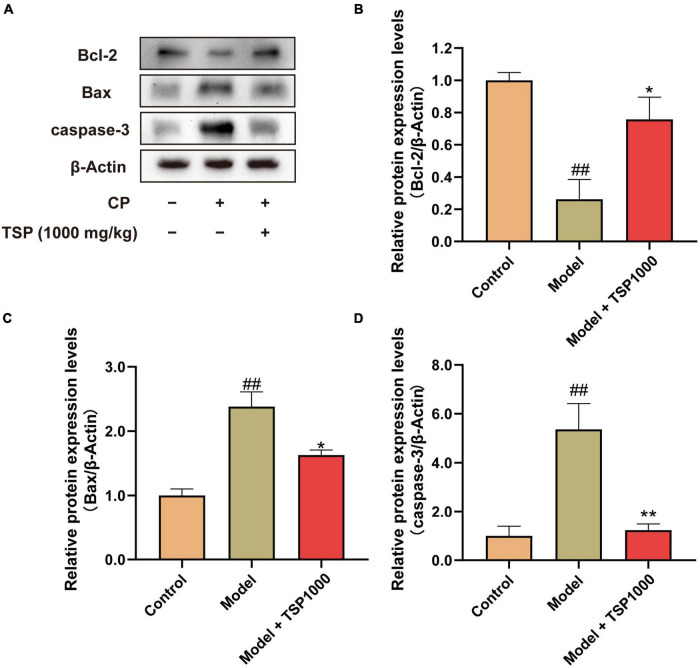
The effects of TSP on the Bcl/Bax/caspase-3 apoptosis pathway. **(A)** The representative images of western blotting results. **(B–D)** Quantification of the protein expression of Bcl-2, Bax, and caspase-3. All data were expressed as mean ± SEM (*n* = 3). ^##^*P* < 0.01 vs. Control group; *P < 0.05, **P < 0.01 vs. Model group.

The Bcl/Bax/caspase-3 apoptosis pathway was detected by the Western blotting experiment (Figure 9). Compared with that of the Control group, CP induced a considerable reduction in Bcl-2 expression (*P* < 0.01, Figure 9B) and a significant increase in Bax and caspase-3 (*P* < 0.01, Figures 9C,D). After 1000 mg/kg TSP administration, the apoptosis pathway-related protein markers were dramatically rescued in the hippocampus (*P* < 0.05, *P* < 0.01, Figures 9B–D).

## Discussion

The anxiety- and depression-like behavior of cancer patients following chemotherapy affects the mental and physical health of patients gravely (28). Nutritherapy is one of the vital approaches for preventing and intervening with anxiety- and depression-like behavior following chemotherapy. In this study, TSP effects on anxiety- and depression-like behavior after CP treatment were explored. Behavior experiments indicated that TSP reversed CP-induced anxiety- and depression-like behavior in mice, and the most effective dose of TSP was 1000 mg/kg/d. In this research, the mechanisms underlying the impact of TSP on anxiety- and depression-like behavior were further investigated using mice administered 1000 mg/kg/d of TSP.

The hippocampus is part of the limbic system of the brain, which is used to translate emotions and record memories. The hippocampus also plays a negative feedback regulation role in the hypothalamic-pituitary-adrenal axis (HPA) that controls stress responses (29). Growing evidence shows that oxidative stress causes oxidative damage to lipids, proteins, and DNA, ultimately hindering the central nervous system (CNS) (30, 31). CP has been proven to cause oxidative stress and elevates ROS in the hippocampus (10). Overproduction of ROS promoted lipid peroxidation and decreased the activity of antioxidant enzymes have been reported in depression (32). Antioxidant enzymes are indispensable for neutralizing extra free radicals in anxiety- and depression-like behavior (33, 34). During oxidative stress, the increase in MDA levels reflects the acceleration of lipid oxidation, which is the end-product of lipid peroxidation (35). In this study, The SOD activity was dramatically decreased, and the MDA levels were obviously increased in the hippocampus. The TSP management reversed these phenomena. In addition, we found that TSP reduces the activity of GSH-Px, which may point out the consumption of GSH-Px to eliminate extra ROS. Moreover, Oxidative stress is positively correlated with the expression of Nrf2, which is widespread in the CNS (36). The high expression of Nrf2 plays a neuroprotective effect by activating antioxidant responsive element (ARE), up-regulating to the transcription of antioxidant proteins such as SOD, GSH-Px, and HO-1 (37). In this study, the expression of Nrf2 and HO-1 were decreased, and the expression of Keap1 was increased in the hippocampus of mice exposed to CP. These abnormal changes were dramatically ameliorated *via* TSP treatment.

Neuroinflammation has been a significant factor in abnormal neurobehavior in neurological diseases (38). Microglial cells are the resident immune cells of the CNS (39). Microglial cells play a pivotal role in maintaining the homeostasis of healthy tissues homeostasis, impacting the neural circuit, and disturbing brain development (40). Under pathological conditions, microglia are activated and transformed into amoebic morphology and act like macrophages, producing and releasing various pro-inflammatory cytokines to recruit peripheral immune cells to cause the neuroinflammatory response (41, 42). The expressions of the Iba-1 and the levels of pro-inflammatory cytokines were tested to assess whether TSP can alleviate CP-induced neuroinflammation in the hippocampus. The data showed that TSP normalized in Iba-1, TNF-α, and IL-1β expressions in the hippocampus of mice, which indicated that TSP could observably ameliorate neuroinflammatory response in the hippocampus of mice.

Oxidative stress and neuroinflammation orchestrate the development of neuronal apoptosis and impaired neurogenesis (43). Here, the *in situ* TUNEL analysis was performed to clarify whether TSP could restore hippocampal neuronal apoptosis. Our data showed that the number of TUNEL-positive neurons in the CA1 and CA3 regions of the Model group was significantly increased vs. that of the Control group. It is revealed that CP could cause neuronal apoptosis. Besides, pro-apoptotic family members and anti-apoptotic family members participate in the regulation of cell apoptosis (44). Eventually, the caspase family of cysteine protease is activated and causes apoptosis protease cascade reaction to irreversible apoptosis (45). Therefore, Western blotting was used to probe the expression of apoptosis markers. We observed that the expression of Bcl-2 (anti-apoptotic protein) in the hippocampus of the TSP group was higher, and the expression of Bax (apoptosis regulator) and caspase-3 (pro-apoptotic protein) was lower vs. that of the Model group.

Moreover, many traditional antidepressants and anxiolytics have been reported to promote neurogenesis in the hippocampus of the DG region (36, 46). In this research, the expression of DCX in model mice was dramatically reduced, consistent with the previous study (47, 48). The expression of DCX in TSP-treated mice was considerably up-regulated. Immunofluorescence results reveal that TSP may promote neurogenesis in the hippocampus, but its underlying molecular mechanism needs further exploration. Neurotrophic factors are essential proteins that play a diversity of physiological functions in the CNS (49). The enhanced neurotrophic factors may benefit neuronal function and play a role in neurogenesis and neuronal survival (50). BDNF is a recognized factor that exerts physiological effects *via* binding with the TRKB receptor (51). CREB is a crucial regulator of neuronal maturation and differentiation in adult hippocampal neurogenesis (52). TSP can restore CP-induced phosphorylate CREB in mice. The results suggest that the BDNF/TRKB/CREB pathway may be involved in the protective effect of TSP to ameliorate CP-induced neuronal apoptosis and neurogenesis, which is consistent with previous papers that the recovery of BDNF function may be the basis of antidepressant therapy (53). Further exploration is needed to expound whether TSP crosses the BBB and the mechanism of its CP-induced anxiety- and depression-like behavior effects.

**FIGURE 10:**
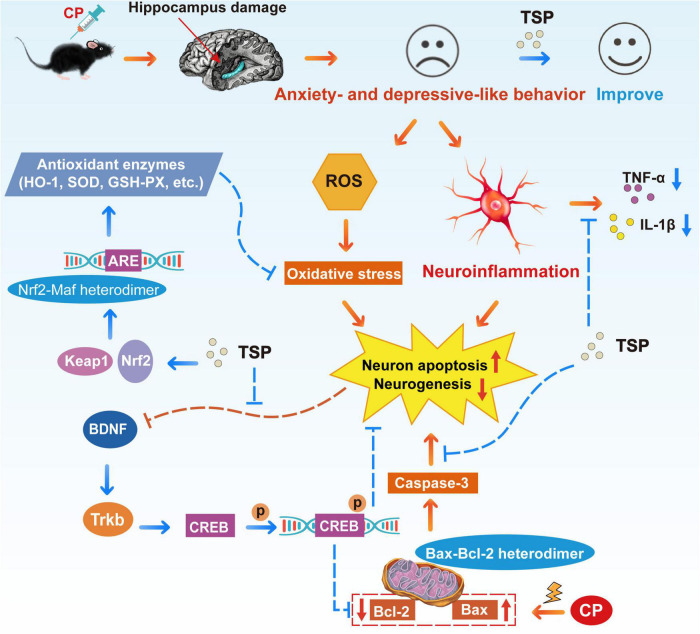
Schematic representation of TSP improving CP-induced anxiety- and depression-like behavior.

In summary, we revealed that TSP effectively reverses CP-induced anxiety- and depression-like behavior in mice. Mechanically, TSP restores oxidative stress, neuroinflammation, neuron apoptosis, and neurogenesis through enhancing the Nrf2/HO-1 and BDNF/TrkB/CREB signaling pathways and regulating the Bcl-2/Bax/caspase-3 apoptosis pathway (Figure 10). TSP might be considered material for developing nutraceuticals for preventing or treating neurobehavioral changes following chemotherapy treatment.

## Data Availability Statement

The original contributions presented in the study are included in the article/supplementary material, further inquiries can be directed to the corresponding authors.

## Ethics Statement

The animal study was reviewed and approved by Animal Ethics Committee of Guangdong Ocean University (approval number: 2019090502).

## Author Contributions

Y-TZ, WW, YL, and SL contributed to the conception and design of the study. HY, CH, JZ, SZ, SC, and WZ performed experiments. HY and ML analyzed the data. Y-TZ, HY, and LJ wrote the manuscript. All authors read and approved the final manuscript.

## Conflict of Interest

The authors declare that the research was conducted in the absence of any commercial or financial relationships that could be construed as a potential conflict of interest.

## Publisher’s Note

All claims expressed in this article are solely those of the authors and do not necessarily represent those of their affiliated organizations, or those of the publisher, the editors and the reviewers. Any product that may be evaluated in this article, or claim that may be made by its manufacturer, is not guaranteed or endorsed by the publisher.
